# Volatile Organic Compounds as Biomarkers of Gastrointestinal Diseases and Nutritional Status

**DOI:** 10.1155/2019/7247802

**Published:** 2019-09-10

**Authors:** Mariangela Rondanelli, Federica Perdoni, Vittoria Infantino, Milena Anna Faliva, Gabriella Peroni, Giancarlo Iannello, Mara Nichetti, Tariq A. Alalwan, Simone Perna, Clementina Cocuzza

**Affiliations:** ^1^IRCCS Mondino Foundation, Pavia 27100, Italy; ^2^Department of Public Health, Experimental and Forensic Medicine, University of Pavia, Pavia 27100, Italy; ^3^Endocrinology and Nutrition Unit, Azienda di Servizi alla Persona “Istituto Santa Margherita”, University of Pavia, Pavia 27100, Italy; ^4^Department of Biomedical Sciences and Human Oncology, University of Bari Aldo Moro, Bari 70121, Italy; ^5^General Management, Azienda di Servizi alla Persona “Istituto Santa Margherita”, Pavia 27100, Italy; ^6^Department of Biology, College of Science, University of Bahrain, Sakhir Campus P.O. Box 32038, Zallaq, Bahrain; ^7^Department of Medicine and Surgery, University of Milano-Bicocca, Milano 20126, Italy

## Abstract

**Purpose:**

The purpose of this review was to identify the best solution for rapid and noninvasive diagnosis and long-term monitoring of patients affected by inflammatory gastrointestinal diseases, colon and gastric cancer, obesity in correlation to diet, and breast milk to evaluate exposure to VOCs in women and infants.

**Methods:**

This review included 20 previously published eligible studies. VOC analysis has allowed us to highlight differences in lifestyles, intestinal microbiota, and metabolism. New innovative methods have been described that allow the detection and quantification of a broad spectrum of metabolites present in exhaled breath even at very low levels, some of which have been shown to be indicators of pathological conditions.

**Results:**

Five studies were analyzed that involved VOC analysis in relation to type of diet. All of them showed that the type of diet can have an impact on metabolites excreted and therefore can be a useful tool in the nutritional studies related to metabolism and health and disease status. Two studies concerned VOC analysis in inflammatory bowel diseases, and the results showed that VOCs can distinguish active disease from remission; VOC profile is clearly different in patients. In particular, C_15_H_30_ 1-pentadecene, 3-methyl-1-butanal, octane, acetic acid, alpha-pinene, and m-cymene are elevated in active ulcerative colitis. Four studies examined VOCs in gastric and colorectal tumors showing a change in metabolic biomarkers of cancer patients compared to the control group. Finally, the study of VOCs in breast milk has improved the understanding of the potential health risks of exposure of children to chemical pollutants.

**Conclusions:**

VOC analysis allowed to highlight differences in behavior, lifestyle, and metabolism of individuals. Analytical methods are continuously developed to allow for better detection and quantification of metabolites, thus enabling the detection of a broader spectrum of pathophysiology and disease biomarkers.

## 1. Introduction

Intermediate products of metabolism are considered biomarkers specific to human clinical and nutritional status and therefore can be a useful diagnostic support to monitoring metabolic disease, including chronic inflammatory disease and gastrointestinal disease, using noninvasive methods. In fact, it is possible to construct specific metabolic profiles for volatile organic compounds (VOCs) with low-molecular weight, which are divided into the gaseous phase by the alveolar blood and appear in the exhaled breath. Breath analysis is a noninvasive and sensitive medical diagnostic tool and offers the possibility of rapid measurements, thanks to the development of more robust analytical methods that allow the detection of volatile metabolites present in the breath at low concentrations [1]. Breath collection offers a method for assessing VOCs not only in blood but also in feces, milk, saliva, and urine, thus providing information on the state of health of the lung, the gastrointestinal tract, and urinary tract, respectively. The origin of intermediate products is not yet fully understood. Some VOCs present in the breath are not found in the blood, urine, or saliva, so it is thought that some organs are involved in their conversion. In particular, the liver is an organ that can be involved in the conversion of VOCs present in the bloodstream bringing the concentration of VOCs under the detectable level and increasing the concentration of new VOCs in the blood flow. VOCs produced from the fecal matter in the gastrointestinal tract can be transported into the blood and eventually reach the lungs and appear in breath. However, they may not be detected in the breath due to conversion in the liver. In fact, the human liver has an array of highly specific enzymes that are able to oxidize nonpolar compounds to more polar, hydrophilic compounds that are easily removed through the excretory system. In addition to the liver, other organs like the lungs, kidneys, and bladder may have biotransformation activities. Furthermore, evidence of VOCs transformation is also reported in the nose [2].

No single analytical method is sufficient for measuring all the VOCs present in the bodily fluids and in the breath. Gas chromatography by mass spectrometry technique (GC-MS) is the method mainly used; however, other methods including proton-transfer-reaction mass spectrometry (PTR-MS) and selected ion flow tube mass spectrometry (SIFT-MS) are very much used in the characterization of VOCs. Methods involving solvent extraction techniques have been used in studies of VOCs in milk. The GC-MS technique in association with sample collection and concentration techniques such as solid phase microextraction (SPME) and thermal desorption has provided promising results [3]. The reproducibility of SIFT-MS analyses of VOCs had been previously evaluated by determining the variability coefficients for acetone, ammonia, isoprene, propanol, ethanol, acetic acid, and cyanitrate [4–6].

For example, in the study by Martin et al. [4], the SPME technique in combination with GC-MS was used for the analysis of VOCs in samples of human breath without collecting and condensing the exhaled breath. The authors of the study described a new procedure that involves active sampling and preconcentration of exhaled breath vapor (EBV) and respiratory vapor collection with a modified SPME Fiber, mounted within a range of commercial products the RTube™. Immediately after sample collection, at −80°C and at room temperature, the compounds are desorbed from the SPME Fiber in the GC-MS injector at 250°C. The methods were compared in terms of ease of use, speed of analysis, and limits of detection. A supply of clean air was needed for the study subjects, as demonstrated using several localized sources of VOC contaminants including nail polish, lemonade, and gasoline. The authors of the study concluded that the SPME method offers advantages in terms of collection and analysis of EBV, field-portability, elimination of power or cooling requirements, and ability to perform sample collection in a contaminated environment.

Another technique used for the analysis of VOCs is the use of electronic noses (eNoses), which provide fingerprints of VOCs exhaled, called breathprints [7]. It has been shown that the breathprints are modified according to different pathological states such as asthma and chronic obstructive pulmonary disease (COPD). In their study, the authors describe the application of electronic noses in the analysis of exhaled breath, as a rapid and noninvasive diagnostic tool, in the diagnosis and monitoring of chronic diseases of the respiratory tract. Furthermore, they recommended the construction of a database comprising of disease-specific breathprints.

In particular, some authors [8] have recently evaluated the ability of eNose to detect airway inflammation such as chronic obstructive sleep apnea (OSA), a pathology often associated with obesity, which is becoming a world epidemic. The authors demonstrated how using the electronic nose allowed the rapid analysis of VOC spectra. The authors Dragoneri et al. [9] demonstrate in another study that the electronic nose can detect obstructive sleep apnea (OSA) such as chronic obstructive pulmonary disease (COPD). Sometimes, OSA and COPD are associated in the so-called overlap syndrome (OVS). In this pilot study, the authors hypothesize that the electronic nose could discriminate the exhaled breath of OVS patients from that of subjects with OSA and COPD alone. In particular, 13 patients with OSA, 15 patients with COPD, and 13 with OVS participated in a cross-section study. The exhaled breath was collected by a previously validated and sampled method using an electronic nose (Cyranose 320). Raw data were analyzed by canonical discriminant analysis on principal component reduction. Cross-validation accuracy (CVA) and ROC-curves were calculated. Breathprints of patients with clustered OSA distinctly from those with OVS (CVA = 96.2%) as well with those with COPD (CVA = 82.1%). Breathprints from OVS were not significantly separated from those of COPD (CVA = 67.9%). External validation in newly recruited patients (6 OSA, 6 OVS, and 6 COPD) was tested using the previous training set. The authors concluded that the electronic nose can accurately distinguish the exhaled VOC profile of patients with OSA from those with OVS and those with COPD. It was also reported that the exhaled breath of obese patients with OSA differed from that of nonobese controls. The study evaluated the influence of obesity on the composition of exhaled VOCs by comparing obese subjects with and without OSA and identified the discriminating VOCs in the two groups. The exhaled breath was collected and analyzed using eNose and GC-MS. The authors highlighted how the presence of OSA altered the pattern of exhaled VOCs in obese subjects. They proposed that the incomplete separation of breathprints may be due to the underlying inflammation.

In the present study, a research was carried out to evaluate how the analysis of VOCs can be used in clinical practice for rapid and early diagnosis and for the long-term monitoring of patients suffering from various diseases, focusing our attention on inflammatory gastrointestinal diseases, colorectal and gastric cancer, obesity in correlation with diet, and breast milk to assess the exposure to VOCs of lactating women and infants.

## 2. Materials and Methods

The present systematic review was performed following the steps by Egger et al. [10] as follows: (1) a working group was configured as follows: three operators skilled in clinical nutrition, of whom one acting as a methodological operator and two participating as clinical operators; (2) the revision question on the basis of considerations made in the abstract was formulated as follows: “the state of the art on role on VOCs in the nutritional field focusing in particular on the analysis of VOCs in gastrointestinal diseases, colon and gastric cancer, breast milk, obesity and correlation with diet,” (3) relevant studies were identified as follows: a research strategy was planned, on PubMed (Public Medline run by the National Center of Biotechnology Information (NCBI) of the National Library of Medicine of (USA)) as follows: (a) definition of the key words (volatile organic compounds, gastrointestinal diseases, colon and gastric cancer, breast milk, obesity, and diet), allowing the definition of the interest field of the documents to be searched, grouped in quotation marks (“. . .”) and used separately or in combination; (b) use of the Boolean AND operator that allows the establishment of logical relations among concepts; (c) research modalities: advanced search; (d) limits: time limits: papers published in the last 30 years; languages: English; (e) manual search performed by the senior researchers experienced in clinical nutrition through revision of reviews and individual articles on VOCs in the nutritional field focusing in particular on the analysis of VOCs in gastrointestinal diseases, colon and gastric cancer, breast milk, obesity, and correlation with diet in published in journals qualified in the Index Medicus; 4. the analysis was carried out in the form of a narrative review of the reports.

## 3. Results

**Figure 1:**
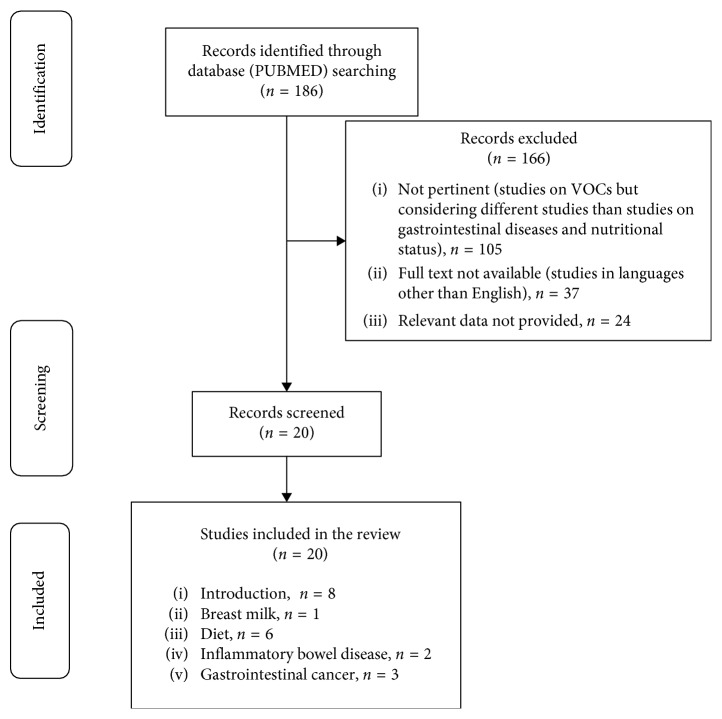
Study selection process.

Figure 1 shows the flowchart of the study selection process, while Table 1 summarizes the studies presented in the narrative review.

### 3.1. Breast Milk

The study by Blount et al. [17] provides an in-depth evaluation of methods for collecting and analyzing human breast milk samples for the presence of VOCs.

This study describes the development and validation of methods for collecting, storing, and analyzing 36 different VOCs in breast milk to evaluate the exposure to VOCs of lactating women and nursing infants. The loss of volatile analyzed was minimized by collecting and storing 3 mL samples in small containers resulting in 70% recovery for all 10 VOCs detected in the majority of breast milk samples collected from 12 women. Potential contamination from chloroform, benzene, toluene, ethylbenzene, xylenes, and methyl-*tert*-butyl ether was reduced to a minimum with the collection materials. The detection of the method was obtained using SPME and selective monitoring of mass spectrometry ions. The authors reported that 10 of the 36 VOCs were detectable in most samples, namely, *m/p*-xilene (0.539 ng/mL), toluene (0.464 ng/mL), 1,4-dichlorobenzene (0.170 ng/mL), tetrachlorethylene (0.165 ng/mL), *o*-xylene (0.159 ng/mL), ethylbenzene (0.0149 ng/mL), styrene (0.129 ng/mL), benzene (0.080 ng/mL), chloroform (0.030 ng/mL), and methyl-*tert*-butyl ether (0.016 ng/mL).

Measuring the levels of VOCs in milk makes it possible to better understand the exposure of infants to chemical pollutants and the potential risk to health. However, the collection and analysis of milk for these compounds are difficult, and robust methods are needed for accurate assessments of child exposure. The presence of lipids is a characteristic of milk that presents many challenges in ensuring an unbiased analysis of VOCs. The relatively high lipid content compared to other biological matrices (e.g., blood and urine) means that milk is more susceptible to contamination through contact with air and laboratory materials commonly used for collection, storage, and analysis of samples. As a result, the collection and storage of samples in sealed containers are important. Furthermore, adequate control samples are crucial for the identification of analytes, including furan, methyl *tert*-butyl ether (MTBE), tetrachloroethylene, and toluene. Using the methods described in this study, a wide range of VOCs can be accurately quantified in human milk.

### 3.2. Diet

Regarding the study of VOCs and the effect of diet on them, some works have been found in the literature. For example, Raninen et al. [12] performed a pilot study on exhaled breath to evaluate the metabolic effects of dietary fiber (DF). In particular, they hypothesized that a diet high in DF containing whole grain may increase the levels of VOCs. In the exhaled breath, 2-methylbutyric acid and 1-propanol decreased at 120 minutes postprandial with the high DF diet, while an increase in ethanol, 1-propanol, acetoin, and propionic acid was observed in patients with the high DF diet. The results of this study suggest that exhaled breath is one matrix used to study the metabolic effects of DF. The high DF rye diet had an effect on the VOCs present in the exhaled breath although the changes in many compounds were individual. Furthermore, by consuming only one meal, an effect was noted on the levels of VOCs, which were indicative of the digestion status of patients, a factor that must be taken into account in the studies on the metabolic effects of nutrients.

Vuholm et al. [11] investigated whether whole-grain wheat (WGW) and whole-grain rye (WGR) improve gut health compared to refined wheat (RW) with the primary outcomes of microbiota composition and gastrointestinal symptoms. The authors reported that the microbiota composition was not affected by diet and that fecal butyrate concentrations decreased in the RW group compared to the WGW and WGR groups. The study concluded that regular consumption of WGR and WGW affected butyrate production and gastrointestinal symptoms and supporting the hypothesis that WGR and WGW can be included in diet equally to maintain gut health. In the same study, respiratory hydrogen was evaluated using a handheld Gastro + Gastrolyzer® (Bedfont Scientific Ltd.) measuring in parts per million (ppm). The subjects were instructed to take a deep breath, hold it for 15 seconds, and exhale steadily in the plastic mouthpiece of the device. Analysis of breath hydrogen concentration was performed during the fasting level and at the peak value, defined as the highest measured hydrogen value within 4 hours of ingesting the disaccharide solution. Subjects were accordingly grouped as hydrogen producers and nonproducers. The findings of the study revealed that fasting level and peak value of breath hydrogen did not differ in any of the diet groups when analyzed as the relative change from baseline to week 6. The number of nonhydrogen producers (subjects with an increase <20 ppm) were equally distributed between diet groups, and excluding the subjects from the analyses did not change the results.

Another study by Baranska et al. [13] examined the profile of VOCs excreted in exhaled breath of 20 healthy individuals over a 13-week period while adhering to a gluten-free diet for 4 weeks before adhering to a normal diet. Thermal desorption gas chromatography combined with time-of-flight mass spectrometry (TD-GC-tof-MS) was used in combination with chemometric analysis to detect a mixture of VOCs in the exhaled breath. Dietary intake was assessed during the study to confirm adherence to the diet and to obtain a better insight into differences in macronutrient intake during the intervention period. A set of 12 VOCs distinguished the samples obtained during the gluten-free diet from those obtained during a normal diet. Seven compounds could be chemically identified (2-butanol, octane, 2-propyl-1pentanol, nonanal, dihydro-4-methyl-2 (3H)-furanone, nonanoic acid, and dodecanal). The results suggest that a gluten-free diet has a reversible impact on the excreted metabolites of the participants as evident in their breath. Several explanations are proposed on the influence of the metabolic state through the diet, although the exact origin of the discriminating compounds is not yet known. The authors managed to demonstrate a new potential use of exhaled air analysis, which represents a useful tool in the fields of nutrition and metabolism.

There are relatively few studies that have highlighted the urinary metabolic signature in relation to excess body weight and obesity. Cozzolino et al. [14] evaluated the urinary VOCs profile of 21 overweight/obese subjects (OW/Ob) and 28 children of normal weight (NW) from the Italian cohort of the I. Family study. Urine samples were analyzed by SPME GC-MS under acid and alkaline conditions to profile a VOC library of urinary metabolites with different physicochemical properties. The authors used multivariate statistical techniques to visualize case clusters and detect VOCs that differentiated OW/Ob from NW children. Fourteen VOCs were identified, in alkaline conditions, which appeared to be pivotal in distinguishing OW/Ob from NW children. The results suggested a difference in the VOCs profile between OW/Ob and NW children. Nevertheless, the authors of the study concluded that the biological and pathophysiologic significance of the observed differences should still continue to be investigated, in order to better understand the potential of urinary VOCs as early metabolic biomarkers of childhood obesity.

Another recent study [15] described a high-throughput quantitative analytical method for the simultaneous measurement of small aliphatic nitrogen biomarkers, i.e., 1,6-hexamethylenediamine (HDA), isophoronediamine (IPDA), p-methylamino-1-alanine (BMAA), and trimethylamine *N*-oxide (TMAO), in human urine. The urinary metabolites, 1,6-hexamethylenediamine (HDA) and isophoronediamine (IPDA), are biomarkers of environmental exposure to their corresponding diisocyanates, while *β*-methylamino-l-alanine (BMAA) is formed as a result of human exposure to food contaminated with blue-green algae. Trimethylamine *N*-oxide (TMAO) is excreted in the urine due to the consumption of diets rich in carnitine and choline, which include animal products and by-products. All of these urinary biomarkers represent classes of small aliphatic nitrogen-containing compounds that have a high aqueous solubility, low logP, and/or high alkaline pKa. Because of their highly polar nature, the analysis of these compounds in complex sample matrices is often challenging. In the aforementioned study, the authors used ultraperformance liquid chromatography-tandem mass spectrometry (UPLC-MS/MS) for simultaneous measurement of VOCs in human urine. Optimization of separation was obtained using heptafluorobutyric acid as a mobile phase and carried out on reversed-phase C18 column. The four analytes were baseline separated within 2.6 minutes with a total run time of 5 minutes, and the detection limits ranged between 0.05 and 1.60 parts per billion (ppb). All samples were acid-hydrolyzed for 4 hours and treated, prior to analysis, by solid-phase extraction (SPE) using a strong cationic sorbent bed with 7N ammonia solution in methanol as eluent. The authors concluded that this method can be applied to determine human exposure to HDI, IPDI, BMAA, and carnitine/choline in population-based studies, such as the National Health and Nutritional Examination Survey (NHANES), in addition to other clinical studies that desire noninvasive urine sampling.

Another similar study was conducted by Alkhouri et al. [16] who used SIFT-MS to evaluate exhaled breath VOCs from OW/Ob children compared to lean children. The results showed differences in concentration of more than 50 compounds between the obese group and the lean controls. The authors identified four VOCs, namely, isoprene-1decene, 1-octene, ammonia, and hydrogen sulphide, that were significantly higher in the obese group compared to lean the group. They concluded that obese children have a unique pattern of exhaled VOCs, which can be utilized in the screening of obesity-related comorbidities, as well as gaining insight into the process and pathways leading to the development of childhood obesity.

### 3.3. Inflammatory Bowel Disease

Smolinska et al. [18] evaluated the potential of VOCs for detecting active disease in patients with ulcerative colitis (UC). Current practice involves the endoscopic assessment of mucosal inflammation to optimise treatment of UC. Nevertheless, simple, inexpensive, and effective tool for the assessment of mucosal inflammation is desirable. Detection of exhaled VOCs may serve as a noninvasive approach for Chron's disease diagnosis. In their study, the authors concluded that VOCs can accurately distinguish active disease from remission in UC, and profiles in UC were distinctly different from profiles in non-UC patients. In particular, C_15_H_30_ pentadecene, 3-methyl-1-butanol, octane, acetic-acid, alfa-pinene, and m-cymene were elevated in the active UC group.

Another study [19] evaluated fecal VOCs in healthy donors and patients with gastrointestinal disease. The authors of that study hypothesized that VOCs would be shared in health; VOCs would be constant in individuals; and specific changes in VOCs would occur in the disease. VOCs by the asymptomatic donors were analyzed and identified by SPME GC-MS in a cohort and in a longitudinal study. Subsequently, the fecal VOCs found in the cohort study were compared with that found in patients with ulcerative colitis, *Campylobacter jejuni* and *Clostridium difficile*. A total of 297 VOCs were identified in the cohort study, among them, ethane, butanoic, pentanoic acid, benzaldehyde, ethanol, carbon disulfide, dimethyldisulphide, acetone, 2-butanone, 2,3-butanedione, 6-methyl-5-hepten-2-one, indole, and 4-methylphenol were present in all the samples. Moreover, the results showed that the majority of donors (80%) shared 44 VOCs in the cohort study. In the longitudinal study, 292 volatiles were identified, with some inter- and intradonor variations of VOC concentrations over time. In comparison to healthy donors, the fecal VOCs of patients with UC, *C. difficile*, and *C. jejuni* were reported to be significantly different [19].

### 3.4. Gastrointestinal Cancer

Recently, several studies have focused on the connection between the composition of specific VOCs in exhaled breath and various forms of cancer. In particular, a study by Wang et al. [20] on 16 colorectal cancer (CRC) patients showed a change in metabolic biomarkers compared to the control group. CRC patients were reported having lower levels of phenyl methylcarbamate, ethylhexanol, and 6-t-butyl-2,2,9,9-tetramethyl-3-5-decadien-7-yne, while having high levels of 1,1, 4,4,-tetramethyl-2,5-cyclohexane-dimethilene. The analysis of blood VOCs appears to have clinical applications for CRC patients.

Another study [23] investigated whether patients with colorectal cancer have a specific VOC compared with the healthy population. Exhaled breath was collected in an inert bag from patients with colorectal cancer and healthy controls and processed offline by thermal-desorber gas chromatography-mass spectrometry to evaluate the VOC profile. During the trial phase, VOCs of interest were identified and selected, and VOC patterns able to discriminate patients from controls were set up; in the validation phase, their discriminant performance was tested on blinded samples. A probabilistic neural network (PNN) validated by the leave-one-out method was used to identify the pattern of VOCs that better discriminated between the two groups. 37 patients and 41 controls were included in the trial phase. Application of a PNN to a pattern of 15 compounds showed a discriminant performance with a sensitivity of 86 per cent, a specificity of 83 per cent and an accuracy of 85 per cent. The pattern of VOCs in patients with colorectal cancer was different from that in healthy controls. The PNN in this study was able to discriminate patients with colorectal cancer with an accuracy of over 75 per cent. Breath VOC analysis appears to have potential clinical application in colorectal cancer screening.

Two further studies highlighted the correlation between VOCs and gastric cancer (GC). Leja et al. [21] demonstrated that although VOCs measurements were well reproducible in GC patients, and specific modifications of the intestinal microbiome may have influenced the VOC results. The authors concluded that gastrointestinal interventions, including the use of antibiotics in *Helicobacter pylori* eradication and bowel cleansing for colonoscopy, could potentially affect the diagnostic accuracy of breath VOCs. The other study by Kumar et al. [22] used SIFT-MS to monitor the VOCs in the exhaled breath of 81 patients with esophageal or gastric adenocarcinoma and 129 healthy controls. VOC levels of pentanoic acid, hexanoic acid, phenol, methyl phenol, ethyl phenol, butanal, pentanal, hexanal, heptanal, octanal, nonanal, and decanal were reportedly to be significantly higher in the cancer group compared to the noncancer controls (*P* < 0.05). In addition, the authors of the study were able to distinguish patients with esophageal and gastric adenocarcinoma from healthy control subjects through their specific VOCs profiles.

## 4. Discussion

This narrative review of the recently published literature indicates that breath analysis is a noninvasive method for assessing individuals' health or disease status and provides support for clinical trials, diagnosis, and therapeutic monitoring. The exhaled breath is a complex mixture of low-molecular weight VOCs that are derived from the diet and endogenous metabolism or from the microbiota of the gastrointestinal and respiratory tracts. Metabolic, inflammatory, and neoplastic conditions are associated with characteristic respiratory profiles, and breath analysis has been identified as a potentially simple and noninvasive method for the screening and monitoring of pathological or metabolic disorders such as asthma, diabetes mellitus, obesity, and cancer. Compared to the study of the subjects' health status, with traditional biomarkers, the analysis of VOCs reflects the individual's fingerprint and represents an example of personalized medicine. However, there are a number of factors that influence the concentrations of VOCs in the exhaled air, including diet, nutritional status, physical activity, and smoking habits. Analysis of VOCs allows to highlight the differences in behaviour and lifestyle habits and variations in the metabolism of individuals. Analytical methods are continuously being improved which allows for better detection and quantification of the metabolites. This, in turn, will allow for the identification of a wide spectrum of biomarkers present in exhaled breath even at very low levels, some of which may be indicators of pathologies. In order to improve this research, it is necessary to give greater emphasis to the identification and quantification of disease biomarkers through further development of more sensitive analytical methods capable of analyzing exhaled breath in real time and avoiding contamination by exogenous compounds. Furthermore, to apply breath analysis to studies in human nutrition, it is imperative to consider any concomitant comorbidity, and breath sampling should take place under standardized conditions, for example, after an overnight fast with daytime monitoring.

## Figures and Tables

**Table 1:** Studies presented in the narrative review.

Authors/year	Type of study	Aims	Sample	Methods	Duration	Endpoint and results
*Diet*						
Vuholm et al. [11]/2017	Randomized parallel trial	The authors investigate whether WGW and WGR improve gut health in different ways compared to RW, with the primary outcomes of microbiota composition and gastrointestinal (GI) symptoms	70 healthy adults (in means 6 SDs; aged 51.0–69.4 y, body mass index (BMI (in kg/m^2^)) 27.8–61.9, 32 : 38 men : women)	Healthy adults replaced cereal foods from their habitual diet with WGR, WGW, or RW; before and after a 6-wk intervention, the stool sample was collected and analyzed for short-chain fatty acids and microbiota composition use of 16S ribosomal RNA gene-targeted high-throughput amplicon sequencing	6-week intervention	Intakes of whole grains were 145.2 6 75.9, 124.2 6 57.3, and 5.4 6 3.2 g/d in the WGW, WGR, and RW groups, respectively. Gut microbiota composition was not affected by diet. The relative change in fecal butyrate decreased in the RW (238%) group compared to the WGW (25%, *P*=0.004) and WGR groups (21%, *P*=0.037). Other short-chain fatty acids were unaffected.
Raninen et al. [12]/2016	Article original research	The aim was to examine the potential of exhaled breath analysis to study the metabolic effects of DF	7 men	Alveolar exhaled breath samples were analyzed at fasting state and 30, 60, and 120 minutes after this meal parallel to plasma glucose, insulin, and serum lipids; we used solid-phase microextraction and gas chromatography-mass spectrometry for detecting changes in 15 VOCs	30, 60, and 120 minutes after this meal	Exhaled breath 2-methylbutyric acid in the fasting state and 1-propanol at 120 minutes decreased (*P*=0.91 for both) after an HFD. Ingestion of the test meal increased ethanol, 1-propanol, acetoin, propionic acid, and butyric acid levels while reducing acetone, 1-butanol, diacetyl, and phenol levels. Both DF diet content and having a single meal affected breath VOCs.
Baranska et al. [13]/2013	Longitudinal study	Follow volatile organic compounds excreted in exhaled breath of individuals while adhering to a gluten-free diet for 4 weeks prior to adherence to a normal diet	20 participants without any known food intolerance (female: 12, male: 9, age 16–61 years (average 36 years), BMI 18.9–35.3 (average 24)	TD-GC-tof-MS in combination with chemometric analysis to detect an array of VOCs in exhaled breath; dietary intake was assessed to verify adherence to the diet and to get insight into macronutrient intake during the intervention period	4 weeks	The study shows that the composition of VOCs in exhaled breath changes as a result of a dietary intervention, a gluten-free diet. The paper share a new potential use of exhaled air analysis and might become a useful tool in fields of nutrition and metabolism.
Cozzolino et al. [14]/2017	Family cohort	The aim of this study was to evaluate the VOCs profile in the urine	21 OW/Ob (10 females and 11 males, age 12.4 ± 1.2 years, BMI 26.7 ± 4.2 kg/m^2^) and 28 NW (16 females and 12 males, age 12.9 ± 1.5 years, BMI 19.5 ± 1.8 kg/m^2^)	Urine samples were analyzed by SPME-GC-MS under both acidic and alkaline conditions, in order to profile a wider range of urinary volatiles with different physicochemical properties; multivariate statistics techniques were applied to bioanalytical data to visualize clusters of cases and detect the VOCs able to differentiate OW/Ob from NW children	6 years	Results suggest that VOCs signatures differ between OW/Ob and NW children; the levels of 2-pentanone, 3-hexanone, 5-methyl-3-hexanone, 4-methyl-2-heptanone, 3-octanone, 2,4,4-trimethyl-1-pentanol, 1-hexanol, 2-hexanol, 1-heptanol, dimethyl sulfone, 2,4,6-trimethyl-pyridine, and formamide *N*,*N*-dibutyl are higher in the urine of OW/Ob children than in NW. In contrast, 1 H pyrrole-2-methyl and 1-methyl-2-piperidone have a lower concentration in OW/Ob children compared to NW.
Bhandari et al. [15]/2018	Article	This work describes a quantitative high-throughput analytical method for the simultaneous measurement of small aliphatic nitrogenous biomarkers, in human urine	Human urine	The authors report on the development of (UPLC-ESI-MS/MS) method for the simultaneous measurement of these biomarkers in human urine; chromatographic separation was optimized using heptafluorobutyric acid- (HFBA-) based mobile phase and a reversed-phase C18 column		This method can be implemented to assess human exposure to HDI, IPDI, BMAA, and carnitine/choline in population-based studies, such as the nationalHealth and nutritional examination survey (NHANES) as well as other clinical studies that desire noninvasive urine sampling.
Alkhouri et al. [16]/2015	Article	The objective of this study was to investigate changes in volatile organic compounds (VOCs) in exhaled breath in overweight/obese children compared to their lean counterparts	Overweight and obese children between the ages of 6 to 18 years were recruited as healthy controls (6–18 years of age)	The exhaled breath samples gas analysis was performed by SIFT-MS on a VOICE200® SIFT-MS instrument		Compared to the lean group, the obese group was significantly older (14.1 ± 2.8 vs. 12.1 ± 3.0 years), taller (164.8 ± 10.9 vs. 153.3 ± 17.1 cm), and more likely to be caucasian (60% vs. 35.2%); *P* < 0.05 for all. A comparison of the SIFT-MS results of the obese group to the lean group revealed differences in concentration of more than 50 compounds. A panel of four VOCs can identify the presence of overweight/obesity with excellent accuracy. Further analysis revealed that breath isoprene, 1- decene, 1-octene, ammonia, and hydrogen sulphide were significantly higher in the obese group compared to lean group (*P* value < 0.01 for all). Obese children have a unique pattern of exhaled VOCs.

*Breast milk*						
Blount et al. [17]/2010	Paper	The authors describe the development and validation of methods for collecting, storing, and analyzing 36 volatile organic compounds (VOCs) in breast milk to assess VOC exposure of lactating women and nursing infants	Aliquots (3 mL) of breast milk collected from 12 women at 30 days postpartum	Milk was collected by one of three methods: 1, Manual expression directly into a vacutainer;2, manual expression into a 30 mL glass jar then immediate transfer into *a* vacutainer; 3, Breast-pump (Hollister, Inc., Libertyville, IL) expression then immediate transfer into a vacutainer; the method used in this study was SPME-GC-MS	10 weeks	Analysis of 12 breast-milk specimens revealed varying levels of 36 different analytes; using the methods described in this paper, a broad range of VOCs can be accurately quantified in human milk.

*Inflammatory bowel disease*						
Smolinska et al. [18]/2017	Paper	The aim of this study was to investigate whether VOCs are able to differentiate between active UC and UC in remission and non-UC colitis	72 UC (132 breath samples; 62 active; 70 remission) and 22 non-UC	Clinical activity index, blood, fecal, and breath samples were collected at each out-patient visit;non-UC colitis was confirmed by stool culture or radiological evaluation;breath samples were analyzed by gas chromatography time-of-flight mass spectrometry and kernel-based method to identify discriminating VOCs	15 months	Eleven VOCs predicted active vs. Inactive UC in an independent internal validation set with 92% sensitivity and 77% specificity (AUC 0.94). Non-UC colitis patients could be clearly separated from active and inactive UC patients with principal component analysis.
Garner et al. [19]/2007	Report	The aim of this work was to define the volatiles emitted from the feces of healthy donors and patients with gastrointestinal disease	30 asymptomatic donors (aged 20–65 y, 15 male), 10 asymptomatic donors (aged 23–65 y, 5 male with ulcerative colitis (*n* 18)	Volatiles from feces were collected by solid-phase microextraction and analyzed by gas chromatography/mass spectrometry	Analyses were undertaken within 7 d of freezing	In the cohort study, 297 volatiles were identified. In all samples, ethanoic, butanoic, pentanoic acids, benzaldehyde, ethanal, carbon disulfide, dimethyldisulfide, acetone, 2-butanone, 2,3-butanedione, 6-methyl-5-hepten- 2-one, indole, and 4-methylphenol were found. Forty-four compounds were shared by 80% of subjects. In the longitudinal study, 292 volatiles were identified, with some inter- and intrasubject variations in VOC concentrations with time. When compared to healthy donors, Volatile patterns from feces of patients with ulcerative colitis, *C. difficile*, and *C. jejuni* were each significantly different.

*Gastrointestinal cancer*						
Wang et al. [20]/2014	Study	The aim of this study was utilize gas chromatography/mass spectrometry (GS/MS) and multivariate analysis to compare the VOCs in blood samples from individuals in a healthy physiological state with the VOCs in blood samples from CRC patients in a phathological state thereby allowing for the identification of CRC specific VOCs in the blood	16 colorectal cancer patients and 20 healthy controls	SPME/GS-MS was used to analysis the exhaled VOCs; the statistical methods principal component analysis (PCA) and partial least-squares discriminant analysis (PLSDA) were performed to deal with the final data		Three metabolic biomarkers were found at significantly lower levels in the group of CRC patients than in the normal control group (*P* < 0.01): Phenyl methylcarbamate, ethylhexanol, and 6-t-butyl-2,2,9,9-tetramethyl-3,5- decadien-7-yne. In addition, significantly higher levels of 1,1,4,4-tetramethyl-2,5-dimethylene-cyclohexane were found in the group of CRC patients than in the normal control group (*P* < 0.05). Compared with healthy individuals, patients with colorectal adenocarcinoma exhibited a distinct blood metabolic profile with respect to VOCs.
Leja et al. [21]/2016	Paper	The objective was to assess the reproducibility of VOCs in gastric cancer (GC) and the effects of conditions modifying gut microbiome on the test results	Ten patients with GC; 17 patients before and after *H. pylori* eradication therapy; 61 patients before and after bowel cleansing	The samples were analyzed by (1) gas chromatography linked to mass spectrometry (GC-MS), applying the nonparametricWilcoxon test (level of significance *P* < 0.05); (2) by cross-reactive nanoarrays combined with pattern recognition	Was sampled for volatile markers for three consecutive days	Exhaled VOCs profiles were stable for GC patients over a three-day period. Alpha pinene m (*P*=0.028) and ethyl acetate (*P*=0.030) increased after the antibiotic containing eradication regimen; acetone (*P*=0.0001) increased following bowel cleansing prior to colonoscopy.
Kumar et al. [22]/2015	Original article	The present study assessed whether exhaled breath analysis using selected ion flow tube Mass spectrometry could distinguish esophageal and gastric adenocarcinoma from noncancer controls	81 patients with esophageal (*N* = 48) or gastric adenocarcinoma (*N* = 33) and 129 controls including Barrett's metaplasia (*N* = 16), benign upper gastrointestinal diseases (*N* = 62), or a normal upper gastrointestinal tract (*N* = 51)	Flow tube Mass spectrmetry instrument was used for analysis of volatile organic compounds (VOCs) within exhaled breath samples; all study participants had undergone upper gastrointestinal endoscopy on the day of breath sampling	2 years	12 VOCs—pentanoic acid, hexanoic acid, phenol, methyl phenol, ethyl phenol, butanal, pentanal, hexanal, heptanal, octanal, nonanal, and decanal—were present at significantly higher concentrations (*P* < 0.05) in the cancer groups than in the noncancer controls. Distinct exhaled breath VOC profiles can distinguish patients with esophageal and gastric adenocarcinoma from noncancer controls.
Altomare et al. [23]/2013	Prospective observational study designed in two phases.	The aim of the initial trial phase was to identify and select VOCs of interest and to set up a VOC pattern potentially capable of discriminating between patients with colorectal cancer and normal controls using an appropriate statistical model; the aim of the subsequent validation phase was prospectively to validate the model in a blinded fashion on a further series of patients and healthy controls; these subjects were not included in the previous phase	The patients with colorectal cancer had histologically proven disease and were admitted to the surgicalDepartment; healthy controls were chosen from patients undergoing screening colonoscopy and found to be disease-free	Exhaled breath was collected in an inert bag from patients with colorectal cancer and healthy controls (negative at colonoscopy) and processed offline by thermal-desorber gas chromatography-mass spectrometry to evaluate the VOC profile; during the trial phase, VOCs of interest were identified and selected, and VOC patterns able to discriminate patients from controlswere set up; in the validation phase, their discriminant performance was tested on blinded samples; a Probabilistic neural network (PNN) was used to identify the pattern of VOCs	6 months	Application of a PNN to a pattern of 15 compounds showed a discriminant performance with a sensitivity of 86 per cent, a Specificity of 83 per cent and an accuracy of 85 per cent (area under the receiver operating characteristic (ROC) curve 0·852). The accuracy of PNN analysis was confirmed in the validation phase on a further 25 subjects; the model correctly assigned 19 patients, giving an overall accuracy of 76 per cent.

*Introduction*						
Španěl and Smith [1]/2011	Review	Summarizes the recent progress made in noninvasive monitoring of volatile compounds in exhaled breath and above biological liquids, as they are becoming increasingly important in assessing the nutritional and clinical status and beginning to provide support to conventional clinical diagnostics and therapy				The significance of the following breath gases and their concentrations are reported: acetone and the influence of diet; ammonia confirmed as an indicator of dialysis efficacy; hydrogen and the 13CO_2_/12CO_2_ ratio (following the ingestion of 13C-labeled compounds) as related to gastric emptying and bowel transit times; hydrogen cyanide released by Pseudomonas and its detection in breath of children with cystic fibrosis; and multiple trace compounds in breath of patients with specific pathophysiological conditions and ‘metabolic profiling.
de Lacy Costello et al. [2]/2014	Topical review	Compendium of all the volatile organic compounds (VOCs) emanating from the human (the volatolome) is for the first time reported	1840 VOCs have been assigned from breath (872), saliva (359), blood (154), milk (256), skin secretions (532) urine (279), and feces (381)			The authors' intention that this database will not only be a useful database of VOCs listed in the literature but will stimulate further study of VOCs from healthy individuals. Establishing a list of volatiles emanating from healthy individuals and increased understanding of VOC metabolic pathways is an important step for differentiating between diseases using VOCs.
Dragonieri et al. [8]/2015	Paper	Assess the influence of obesity in the composition of exhaled VOCs by comparing obese subjects with and without OSA and identify the discriminant VOCs in the two groups	19 obese patients OSA (OO; age 51.2 ± 6.8; body mass index (BMI) 34.3 ± 3.5); 14 obese without OSA (ONO; age 46.5 ± 7.6; BMI 33.5 ± 4.1); 20 nonobese healthy controls BMI 24.9 ± 3.8)	Exhaled breath was collected and sampled by using an electronic nose (cyranose 320) and by gas chromatography-mass spectrometry (GC-MS) analysis	10 days	Analysis of collected breath samples enabled the identification of 49 volatile organic compounds that characterized the two investigated groups.
Fens et al. [7]/2015	Review	This review describes the current status on clinical validation and application of breath analysis by electronic noses in the diagnosis and monitoring of chronic airways diseases		Electronic noses (eNoses) is used to measuring the spectrum of VOCs		The past 20 years paved the way for application of advanced electronic noses in medical practice. Several proof of concept studies have shown promising results for diagnosing different (airway) diseases. Currently, the biggest limitation to progress in the field of exhaled breath diagnostics is the lack of sensors that can be produced identically in large quantities.
Fuchs et al. [6]/2010	Observational investigation without any intervention.	The authors applied solid-phase microextraction onfiber-derivatization (SPME-OFD) to determine aldehyde concentrations in exhaled breath of cancer patients, smokers, and healthy volunteers; this study was intended to find out whether lung cancer could be recognized from aldehydes in the breath of cancer patients	36 subjects	Alveolar breath samples were collected under control of expired CO_2_; reactive aldehydes were transformed into stable oximes by means of on-fiber-derivatization (SPME-OFD); aldehyde concentrations in the ppt and ppb level were determined by means of gas chromatography-mass spectrometry (GC-MS)		Acetaldehyde, propanal, butanal, heptanal, and decanal concentrations showed no significant differences for cancer patients, smokers and healthy volunteers. Exhaled pentanal, hexanal, octanal, and nonanal concentrations were significantly higher in lung cancer patients than in smokers and healthy controls. Lung cancer patients could be identified by means of exhaled pentanal, hexanal, octanal, and nonanal concentrations. Exhaled aldehydes reflect aspects of oxidative stress and tumor-specific tissue composition and metabolism.
Martin et al. [4]/2010	Original paper	SPME was applied, in conjunction with gas chromatography-mass spectrometry to the analysis of volatile organic compounds (VOCs) in human breath samples without requiring exhaled breath condensate collection; an EBV collection with a is described	4 healthy, nonsmoking adults	After sample collection, compounds are desorbed from the SPME fiber at 250°C in the GC-MS injector; experiments were performed using EBV collected at −80 °C and at room temperature, and the results were compared to the traditional method		It is demonstrated that this active SPME breath-sampling device provides advantages in the forms of faster sample collection and data analysis, apparatus portability, and avoidance of power or cooling requirements, and performance for sample collection in a contaminated environment.
Reynolds et al. [5]/2010	Article	This work describes the first interfacing of a thermal desorption unit to an (ion mobility) IM-MS using an ESI (extractive electrospray ionization) source; The potential of (thermal desorption) TD-ESI-IM-MS for the rapid screening of breath volatiles as an alternative technique to TD-GC/MS is demonstrated	Breath samples were collected from a healthy volunteer using an adaptive sampling technique	Thermal desorption unit has been interfaced to an electrospray ionization-ion mobility-time-of-flight mass spectrometer		The combination of temperature-programmed thermal desorption and ion mobility improved the response of selected species against background ions. Analysis of breath samples resulted in the identification of breath metabolites, based on ion mobility and accurate mass measurement using siloxane peaks identified during the analysis as internal lock masses.
Wzorek et al. [3]/2010	Paper	The present paper deals with the problems that occur with concentration determination of dimethylamine (DMA) and trimethylamine (TMA); these occur in the breath of people suffering from renal disease		The application of solid-phase microextraction (SPME) and thermal desorption (TD) with subsequent measurement by GC-MS for the determination of amines is discussed		For DMA, preconcentration by SPME did not give satisfactory results. TMA may be analyzed using SPME preconcentration with an LOD of 1.5 ppb. Thermal desorption with Tenax as the adsorbing material allows reliable concentration determination for TMA (LOD = 0.5 ppb) and DMA (LOD = 4.6 ppb). DMA cannot be stored reliably in Tedlar bags and longer storage on Tenax (with subsequent TD) does not give good repeatability of results. For TMA, storage can be done on Tenax or in bags, the best results for the latter being achieved with Flex Foil bags.
Dragonieri et al. [9]/2016	Cross-sectional study	The first aim is to assess whether an eNose could discriminate the exhaled breath of patients with overlap syndrome (OVS) from that of subjects with obstructive sleep apnea (OSA) and chronic obstructive pulmonary disease (COPD) alone; the secondary aim is to verify whether these classification are replicated in a set of newly recruited patients as external validation	This study comprised a total of 59 patients; the first group is composed by 13 never-smoking patients with established diagnosis of OSA, without previous treatment with CPAP; the second group consisted of 15 subjects without a history of sleep disturbances and with a well-defined diagnosis of COPD; the third group included 13 individuals with a proven diagnosis of OVS.	Exhaled breath was collected by a formerly validated method and sampled by using an electronic nose (cyranose 320); raw data were analyzed by canonical discriminant analysis on principal component reduction; cross-validation accuracy (CVA) and ROC-curves were calculated. External validation in newly recruited patients (6 OSA, 6 OVS, and 6 COPD) was tested using the previous training set.	From December 2014 to August 2016	Breathprints of patients with OSA clustered distinctly from those with OVS (CVA = 96.2%) as well as those with COPD (CVA = 82.1%); breathprints from OVS were not significantly separated from those of COPD (CVA = 67.9%); external validation confirmed the above findings.
